# Asymmetrical gene flow of the recently delisted passerine black‐capped vireo (*Vireo atricapilla*) indicates source‐sink dynamics in central Texas

**DOI:** 10.1002/ece3.4764

**Published:** 2018-12-10

**Authors:** Samantha S. Hauser, Lauren Walker, Paul L. Leberg

**Affiliations:** ^1^ Department of Biology University of Louisiana at Lafayette Lafayette Louisiana; ^2^ School of Environmental and Forest Sciences University of Washington Seattle Washington; ^3^ Present address: Department of Biological Sciences University of Wisconsin – Milwaukee Milwaukee Wisconsin; ^4^ Present address: National Park Service Yellowstone National Park Mammoth Wyoming

**Keywords:** conservation genetics, gene flow, metapopulation, source sink

## Abstract

Habitat fragmentation can produce metapopulations or source‐sink systems in which dispersal in crucial for population maintenance. Our objective was to investigate connectivity among black‐capped vireo (*Vireo atricapilla*) populations in tandem with a demographic study (Biological Conservation, 2016, 203, 108–118) to elucidate if central Texas populations act as a source‐sink system. We genotyped 343 individuals at 12 microsatellite loci to elucidate the movement ecology of the black‐capped vireo in central Texas surrounding Fort Hood; the largest and most stable breeding population of black‐capped vireos inhabit Fort Hood. To gain insight into gene flow among populations, we analyzed genetic differentiation, migration rates, number of migrants, and parentage. We found statistically significant, but low levels of genetic differentiation among several populations, suggesting some limited restriction to gene flow. Across approaches to estimate migration, we found consistent evidence for asymmetrical movement from Fort Hood to the other central Texas sites consistent with source‐sink dynamics. Our results are complementary to black‐capped vireo demographic studies done in tandem showing that portions of Fort Hood are acting as a source population to smaller central Texas populations.

## 1 INTRODUCTION

Habitat loss and fragmentation produce discontinuities in the environment and the distribution of wildlife populations. While habitat loss and fragmentation are often conflated (Fahrig, 2017), here they are considered in concert (hereafter fragmentation) because the two commonly occur together through human development and conversion to agriculture. Fragmentation alters abundance and movement of individuals, and the stability and growth rates of populations (Keyghobadi, 2007). By doing so, fragmentation can be deleterious and increase the risk of edge effects, isolation, inbreeding depression, and probability of extinction (Hagan, VanderHaegen, & McKinley, 1996; Keyghobadi, 2007; Segelbacher et al., 2010). Connectivity among fragmented habitat patches helps to minimize these deleterious effects to species and populations.

Fragmentation can produce metapopulations by leaving behind pockets of seemingly isolated populations that can stabilizing force by providing necessary immigrants to bolster populations (Ewers & Didham, 2006; Hanski & Gilpin, 1991). Metapopulations are groups of interdependent subpopulations that are maintained through an extinction‐recolonization process and are thus dependent on a high degree of connectivity (Hanski, 1998). Source‐sink dynamics, a special type of metapopulation, occur when a source population(s) with net growth supply individuals to sink population(s) that would otherwise decline and eventually go extinct (Brawn & Robinson, 1996; Diffendorfer, 1998; Pulliam & Danielson, 1991). Maintaining connectivity among subpopulations in a source‐sink metapopulation is vital to sustaining sink populations and benefits the whole metapopulation. Sink populations are significant components as they comprise a large portion of the metapopulation, can increase stability of source populations, and increase the genetic variation of the metapopulation (Foppen, Chardon, & Liefveld, 2000; Howe, Davis, & Mosca, 1991). Studying connectivity using molecular markers can inform conservation and help develop management plans necessary to maintain populations of species threatened by fragmentation (Segelbacher et al., 2010).

Genetic markers offer a unique opportunity to infer information about population connectivity independent of traditional methods such as band recovery, radio‐telemetry, GPS tags, etc. that may not be suitable on all study species (Franchini et al., 2017; Moore & Dolbeer, 1989). Population genetic techniques can fill in gaps of knowledge obtained with traditional demography methods and can give additional information about population dynamics and dispersal among populations (Franchini et al., 2017; Kool, Moilanen, & Treml, 2013; Moore & Dolbeer, 1989). In studies of metapopulations and source‐sink dynamics, which are defined by population growth and migration rates among populations, demography, and population genetic approaches can be complementary. Demography methods can provide population growth rates that would be inaccurate with genetic approaches while genetic approaches can infer dispersal via gene flow rates that would be otherwise difficult using demography methods (Peery et al., 2016).

The black‐capped vireo (*Vireo atricapilla*) is a formerly endangered migratory songbird dependent on early successional habitats throughout much of its U.S. breeding range (Grzybowski, Tazik, & Schnell, 1994). Conversion of early‐successional shrub habitat into grazing and other land uses has substantially increased fragmentation across their range (Grzybowski et al., 1994; McFarland, Mathewson, Groce, Morrison, & Wilkins, 2013). Disturbance suppression, especially via fire prevention, has decreased the creation of new early successional habitat, further adding to the fragmentation of the landscape (Grzybowski et al., 1994). Due to fragmentation, the black‐capped vireo has been extirpated from much of their historical breeding range throughout Texas, Oklahoma, and Kansas (Graber, 1961). Now the species is patchily distributed across central and southwestern Texas and southern Oklahoma with Fort Hood, located in central Texas, currently supports the largest concentration of black‐capped vireos (Cimprich & Kostecke, 2006). Widespread brood parasitism by the brown‐headed cowbird (*Molothrus ater)*, a by‐product of fragmentation (Lloyd, Martin, Redmond, Langner, & Melissa, 2013), has significantly decreased black‐capped vireo reproduction, causing major population reductions (Kostecke, & Cimprich, 2008).

Fragmentation across the U.S. breeding range has altered connectivity and population dynamics among black‐capped vireo populations resulting in restricted gene flow (Athrey, Barr, Lance, & Leberg, 2012; Barr et al., 2008; Fazio, Miles, & White, 2004). Populations in central Texas, specifically the region around Fort Hood, have been suggested to function as a source‐sink metapopulation because of fragmentation (Walker, Marzluff, & Cimprich, 2016). Populations on Fort Hood had an overall increasing growth rate, attributed to habitat management and brown‐headed cowbird control, while peripheral central Texas populations had an overall decreasing growth rate, which exhibit high brown‐headed cowbird parasitism rates (Cimprich & Kostecke, 2006; Fazio et al., 2004; Walker et al., 2016).

Only through a combination of ecological and population genetic analyses are we able to elucidate both characteristics of source‐sink dynamics: population growth rates and asymmetrical gene flow. The present study was performed in tandem with a demographic study (Walker et al., 2016) which provided population growth rates, survival, and fecundity for each of our study sites in central Texas and related them to potential demographic drivers such as brown‐headed cowbird control. Our objective was to use genetic markers to investigate connectivity among habitat patches in central Texas near and including Fort Hood. We hypothesized that Fort Hood was the source population for sink populations that are unstable due to lack of brown‐headed cowbird control or habitat management. We predicted that there would be evidence of asymmetric gene flow from Fort Hood to the rest of the central Texas vireo habitat patches, suggesting that source‐sink dynamics may be occurring in central Texas.

## 2 Methods

### 2.1 Sample collection

**Figure 1:**
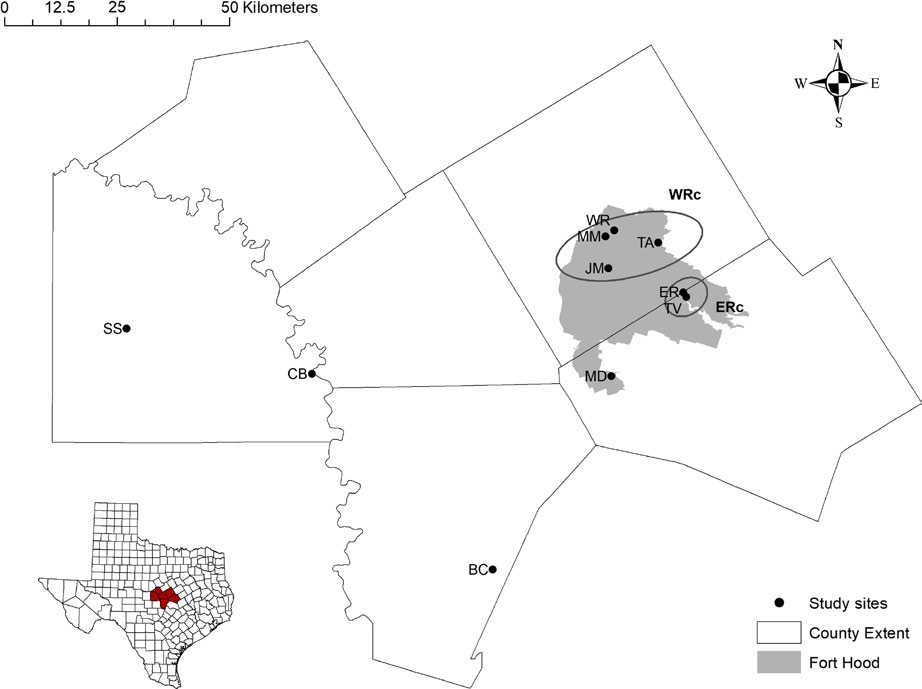
The 10 Black‐capped Vireo study sites on or surrounding Fort Hood military base (depicted in gray): BC: Balcones Canyonlands National Wildlife Refuge; CB: Colorado Bend State Park; ER: East Range; JM: Jack Mountain; MD: Maxdale; MM: Manning Mountain 2; SS: San Saba Property; TA =Training Area 14; TV: Taylor Valley; WR: West Range. Sites encircled in black were combined for analysis resulting in ER_c_ = East Range (Fort Hood) and WR_c_ = West Range (Fort Hood). Inset shows the Texas counties; counties in red are those that encompass the study sites

We collected toenail clips, pin feathers, and blood samples from 343 black‐capped vireos from 10 sites throughout Fort Hood [East Range (ER), Taylor Valley (TV), Jack Mountain (JM), Manning 2 (MM), Maxdale (MD), and West Range (WR)] and the surrounding central Texas habitat patches [San Saba Property (SS), Balcones Canyonlands National Wildlife Refuge (BC), Goldthwaite Property (GP), Colorado Bend State Park (CB)] in central Texas in June of 2014 and 2015 (Figure 1). We captured black‐capped vireos using mist‐nets with black‐capped vireo, white‐eyed vireo (*V. griseus*), or eastern screech owl (*Megascops asio*) song playback. We banded each bird with a unique U.S. Geological Survey band and a unique three color band combination. Birds were aged and sexed using reliable molt limits in the plumage (Pyle, 1997). Collected genetic samples were immediately stored in Queen's Lysis Buffer at 4°C until DNA extraction.

### 2.2 DNA extraction and analysis

We extracted genomic DNA from toenail clip and pin feather samples using the Qiamp Micro DNA Kit Protocol for Isolation of Genomic DNA from Small Volumes of Blood. We genotyped samples at 12 species‐specific microsatellite loci using the primers, *BCVI2‐1, BCVI2‐2, BCVI2‐4, BCVI2‐5, BCVI2‐6, BCVI2‐7, BCVI4‐1, BCVI4‐2, BCVI4‐3, BCVI4‐5, BCVI4‐6, BCVI5–1 *(Barr, Dharmarajan, Rhodes, Lance, & Leberg, 2007). Polymerase chain reaction (PCR) concentrations and cycling conditions from Barr et al. (2007), with the addition of 0.1 mg/ml bovine serum albumin to each sample to increase PCR yield. Each PCR product (1 µl) was added to 9.5 µl of Hi‐Di Formamide (Applied Biosystems) with 0.5 µl of ROX 400HD size standard and run on an ABI 3130 Genetic Analyzer. We scored alleles at each locus using GENEMAPPER software (Thermofisher; v 3.7) and manually checked for error. We ran all homozygotes and an equal number of heterozygotes three times to confirm their genotypes.

We performed tests for deviations from Hardy‐Weinberg equilibrium (HWE), linkage disequilibrium (LD), and null alleles using GENEPOP (v 4.2; Raymond & Rousset, 1995). We excluded samples from populations that deviated from HWE from further analyses reducing our sample size to 338 individuals, as they violate assumptions of downstream analyses. We also used GENEPOP to calculate observed heterozygosity (*H*_o_), expected heterozygosity (*H*_e_), and *F*_IS_ per locus to evaluate genetic diversity in black‐capped vireos at each study site (Raymond & Rousset, 1995). We used the allel.rich function in the hiefstat program in R (v 3.5.0) to calculate allelic richness per locus (*A*_r_), standardized by sample size, for each study site (Goudet, 2005). In these, and in subsequent analyses, sequential Bonferroni corrections were used to adjust alpha levels to control Type I error rates in multiple, related comparisons (Rice, 1989). To determine whether populations differed in genetic diversity, we performed a randomized block ANOVA (R function aov) to test that *A*_r_, *H*_o_, and *H*_e_ did not differ among study sites, blocking by locus, with a post‐hoc Tukey test (R function TukeyHSD) to determine differences between study sites (R Core Team, 2017).

We used several approaches to elucidate population structure among our sampled sites. Population differentiation was estimated with pairwise *F*_ST_ in genepop, with Fisher's exact tests to evaluate significance of differences in allele frequencies. We combined sites on Fort Hood that were not significantly differentiated and adjacent to one another to produce three overall Fort Hood sites. We combined nearby sites with genetically similar composition because treating subsamples of a single population as different populations can bias downstream results. The Fort Hood sites TV and ER were combined to form a new site that we labeled East Range Combined (ER_c_). Fort Hood sites WR, JM, MM, and TA were pooled to for a new site labeled West Range Combined (WR_c_). The last Fort Hood site, MD was left as its own site. The remaining analysis used the resulting six sample sites: SS, BC, CB, ER_c_, MD, and WR_c_. We did not combine Fort Hood sites further as they are separated in space and have a history of being genetically differentiated (Athrey, Barr, et al., 2012; Barr et al., 2008).

Population structure was also assessed using STRUCTURE (v 2.3.4). We used the admixture model with population as a prior in STRUCTURE to determine the number of clusters (*k*) present with our study sites. We evaluated *k* values from 1 to 7, with 10 iterations, 100,000 burn‐in period and 100,000 MCMC (Monte Carlo Markov Chain) repetitions. We used the Puechmaille method to determine *k* in STRUCTURESELECTOR, which accounts for uneven sampling across populations and hierarchical population structure (Li & Liu, 2018; Puechmaille, 2016). We submitted STRUCTURE outputs to CLUMPAK to align clustering results over all runs for a given value of *k* (Kopelman, Mayzel, Jakobsson, Rosenberg, & Mayrose, 2015).

To make inferences about migration patterns among our study sites, we used GENECLASS (v 2.0) and BAYESASS (v 3.0.4). We estimated gene flow directly by first generation migrant detection in GENECLASS. We detected migrants using the Paetkau, Slade, Burden, and Estoup (2004) simulation algorithm and criterion and parameterized the simulations with a 0.01 allelic frequency, 0.01 *p*‐value threshold and 1,000 simulations. We estimated pairwise migration rates between our population in BAYESASS using the program's default settings for parameter estimation (Wilson & Rannala, 2003). BAYEASS can encompass migration between populations beyond first generation migration by using individuals’ ancestral migration. Using both approaches gives us a better understanding of movement among our study sites over a broader time‐scale than is possible using either in isolation.

We used parentage assignment in CERVUS (v 3.0.7) to detect direct migration among populations (Kalinowski, Taper, & Marshall, 2007). After‐second‐year individuals are believed to show strong site fidelity and remain at the same population once their territories are established (Athrey, Lance, & Leberg, 2012). Second‐year individuals go through natal dispersal to establish their breeding territories and are assumed the main dispersers for the species (Pyle, 1997). In CERVUS, we assigned second‐year individuals (*n* = 174) (with a minimum of six loci genotyped) to candidate after‐second‐year parents (*n* = 134) which included 39 candidate mothers and 85 candidate fathers (Kalinowski et al., 2007). We performed simulations of parentage with sexes known based on allele frequencies to assess statistical significance. We used strict (95%) confidence intervals when assessing the parentage assignments. The most likely parent‐offspring pairs were those with the highest likelihood of odds ratio. We identified the parent and their population to indicate the original population of the offspring and to determine if an offspring was a migrant or resident. Second‐year individuals who were found in a different population than their assigned parent (from the after‐second‐year pool) were considered migrants. Those individuals found in the same population as their assigned parent were considered residents.

## 3 RESULTS

**Figure 2:**
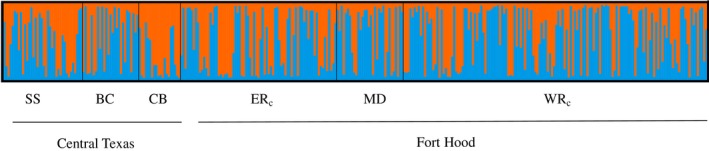
Summary STRUCTURE barplot for 2 clusters (*k*) from 338 individuals across sites: BC: Balcones Canyonlands National Wildlife Refuge; CB: Colorado Bend State Park; ER_c_: East Range (Fort Hood); MD: Maxdale (Fort Hood); SS: San Saba Property; WR_c_: West Range (Fort Hood). Each line represents the genetic signature of an individual with colors representing each cluster

We sampled 343 individuals at our 10 study sites over the 2014 and 2015 summers (Table 1). There were no deviations from HWE after a sequential Bonferroni correction, except at the GP site, which may have been due to a small sample size (*n* = 5) and were excluded from further analyses. None of the locus pairs were out of LD for any population following a sequential Bonferroni correction. There were no significant differences among populations as assessed by estimates of *H*_e_, *H*_o_, and *A*_r_ (*p* > 0.05; Table 1). The overall *F*_ST_ value across our six sites was 0.005 (*p* < 0.001). We found nine pairs of populations to be significantly differentiated after a sequential Bonferroni correction (Table 2). Seven of the nine significantly differentiated population pairs were between Fort Hood and central Texas sites. On Fort Hood, only ER_c_ and WR_c_ were significantly differentiated; however, this result may have been due to the relatively large sample sizes for this comparison as the degree of differentiation was small. Using the Puechmaille method of evaluating *k* from the STRUCTURE output, we identified two genetically distinct clusters in our study system. However, the summary barplot from STRUCTURE assignment probabilities showed no subdivision and considerable admixture among our study sites (Figure 2).

**Table 1:** Summary of sample size, expected heterozygosity (*H*_e_), observed heterozygosity (*H*_o_), and allelic richness (*A*_r_) over 12 loci. Standard errors are in parentheses. There were no significant differences among populations based on a Tukey test with a type I error rate of 0.05

Pop	*n*	*H*_e_	*H*_o_	*A*_r_
SS	76	0.38 (0.018)	0.33 (0.015)	10.06 (1.14)
BC	54	0.37 (0.012)	0.35 (0.016)	8.81 (0.84)
CB	40	0.36 (0.034)	0.32 (0.036)	9.14 (0.93)
ERC	150	0.38 (0.016)	0.33 (0.019)	10.46 (1.14)
MD	64	0.38 (0.016)	0.31 (0.014)	9.64 (1.05)
WRC	292	0.39 (0.011)	0.34 (0.013)	9.99 (1.15)
*p*‐value	pop	0.787	0.803	0.74
	locus	<0.0001	0.93	<0.0001

Note

BC: Balcones Canyonlands National Wildlife Refuge; CB: Colorado Bend State Park; ER_c_: East Range (Fort Hood); MD: Maxdale (Fort Hood); SS: San Saba Property; WR_c_: West Range (Fort Hood).

**Table 2:** Genetic differentiation between sites sampled for black‐capped vireos. Pairwise *F*_ST_ values are depicted on the lower left and *p*‐values are depicted on the upper right. Values that are significant before and after a sequential Bonferroni correction are italicized and bolded, respectively

	SS	BC	CB	ER_c_	MD	WR_c_
SS	–	<0.001	0.005	0.018	0.461	<0.001
BC	**0.013**	–	<0.001	0.024	0.004	0.002
CB	**0.010**	**0.014**	–	0.003	0.023	<0.001
ER_c_	*0.003*	*0.006*	**0.013**	–	0.521	<0.001
MD	−0.002	**0.009**	*0.011*	0.002	–	0.142
WR_c_	**0.007**	**0.005**	**0.013**	**0.002**	0.003	–

Note

BC: Balcones Canyonlands National Wildlife Refuge; CB: Colorado Bend State Park; ER_c_: East Range (Fort Hood); MD: Maxdale (Fort Hood); SS: San Saba Property; WR_c_: West Range (Fort Hood).

Most black‐capped vireo individuals remained at their putative natal population, based on bayesass estimates. The proportion of individuals that remained in their natal population ranged from 67.6% (SS and MD) to 83.2% (WR_c_; Table 3). In general, Fort Hood sites ER_c_ and WR_c_ have the highest estimated proportions of individuals that did not disperse. These two sites were also the only sites that had estimates of emigration that were much larger than their SEs (Table 3); most of the other estimates of emigration are likely not different from zero. In most cases both ER_c_ and WR_c_ migrants contributed at least 10% of individuals to all other sites, with one exception (from ER_c_ to BC). All other sites contributed <5% of individuals to other sampled populations through migration, with the majority contributing <1% of individuals.

**Table 3:** Migration rates between populations as represented as the fraction of individuals in population i (pop i) from population j (pop j) (BAYESASS). Bolded values represent migration rates within a population, that is, the fraction of individuals that remain in a population. Estimates of migration rates that are twice their standard errors (in parentheses) are italicized indicating that migration rate estimates are significantly >0

pop i	pop j					
	SS	BC	CB	ER_c_	MD	WR_c_
SS	**0.676 (0.009)**	0.008 (0.008)	0.008 (0.008)	*0.187 (0.032)*	0.008 (0.008)	*0.114 (0.030)*
BC	0.010 (0.010)	**0.677 (0.010)**	0.010 (0.010)	0.042 (0.024)	0.010 (0.010)	*0.252 (0.029)*
CB	0.013 (0.013)	0.013 (0.013)	**0.680 (0.013)**	*0.172 (0.035)*	0.013 (0.012)	*0.110 (0.033)*
ER_c_	0.010 (0.008)	0.006 (0.006)	0.012 (0.009)	**0.781 (0.020)**	0.005 (0.005)	*0.185 (0.02)*
MD	0.011 (0.010)	0.009 (0.009)	0.009 (0.009)	*0.148 (0.034)*	**0.676 (0.009)**	*0.148 (0.034)*
WR_c_	0.004 (0.003)	0.003 (0.003)	0.003 (0.003)	*0.156 (0.030)*	0.003 (0.003)	**0.832 (0.030)**

Note

BC: Balcones Canyonlands National Wildlife Refuge; CB: Colorado Bend State Park; ER_c_: East Range (Fort Hood); MD: Maxdale (Fort Hood); SS: San Saba Property; WR_c_: West Range (Fort Hood).

We detected 22 migrants with a *p*‐value <0.01 using GENECLASS2 (Table 4). Most migrants were found on Fort Hood (69.6%), 14 of which were found on WR_c _and ER_c_ (60.9% of total migrants). Central Texas sites had low levels of migrants (<15% across sites); however, migrants comprised a larger proportion of the central Texas populations (2%–8%) compared to that of Fort Hood populations (<1%; Table 4).

**Table 4:** Detected migrants (M), proportion of total migrants detected (%M), estimated abundances (N), and proportion of abundances that are migrants (%N) in each population (GENECLASS2)

Population	M	%M	*N*	%*N*
SS	3	13.0	39	7.7
BC	1	4.3	44	2.3
CB	2	8.7	68	2.9
ER_c_	6	26.1	993	0.6
MD	2	8.7	160	1.3
WR_c_	8	34.8	3,292	0.2

Note

BC: Balcones Canyonlands National Wildlife Refuge; CB: Colorado Bend State Park; ER_c_: East Range (Fort Hood); MD: Maxdale (Fort Hood); SS: San Saba Property; WR_c_: West Range (Fort Hood).

In CERVUS, we assigned 21 offspring to parents, comprised of 15 migrants and six residents, at 95% confidence intervals (Table 5). Most migrants (offspring not found in the same population as their parents) were from Fort Hood (73%). We observed asymmetrical migration from Fort Hood to central Texas, with 47% of the migrations from Fort Hood to central Texas and 13% from central Texas to Fort Hood. The remaining migrations were among Fort Hood (13%) or among central Texas sites (27%). Residents (offspring sampled in the same population as their parents) were mostly from Fort Hood samples (83%).

**Table 5:** Numbers of offspring assigned as Migrants or Residents (N) with unidirectional movement shown between sites on Fort Hood (FH) and central Texas (CT) (e.g., FH to CT indicates migration from FH to CT). Percentage of migrants or residents (%) and of total offspring assigned (% Total) are also shown for comparison

Movement	*N*	%	% Total
Migrants	15	–	71
FH to CT	7	47	33
CT to FH	2	13	10
CT to CT	2	13	10
FH to FH	4	27	19
Residents	6	‐	29
CT	1	17	5
FH	5	83	24

## 4 DISCUSSION

In this study, levels of gene flow were restricted sufficiently to lead to statistically significant, but low levels, of population differentiation between central Texas and Fort Hood sites, but not within Fort Hood. Regardless, there was high exchange of individuals across populations despite the statistically significant structuring. Migration estimates between sampled sites on Fort Hood indicate high levels of gene flow throughout the military base. Three approaches showed asymmetrical migration from Fort Hood to central Texas populations. Although the three analyses, BAYESASS, GENECLASS2, and CERVUS, all estimate different aspects of migration, they consistently showed a pattern of movement from Fort Hood sites ER_c_ and WR_c_ to MD and the other central Texas sites. Specifically, estimated migration indicated that ER_c_ and WR_c _contribute substantial numbers of migrants to the rest of the sampled sites.

Restricted gene flow by black‐capped vireos is due to male philopatry and plays a role in population structuring in central Texas (Athrey, Lance, et al., 2012). A combination of increased abundances (Cimprich & Kostecke, 2006; Noa, Hirth, Donovan, & Cimprich, 2007) and a limited number of territories for black‐capped vireos could explain high gene flow within Fort Hood; as populations reach carrying capacity, more individuals are forced to migrate to new populations. A pattern of higher gene flow within Fort Hood than between other sampled sites could be attributed to close proximity of the former as well as to a fragmented landscape among the latter (Lindsay et al., 2008; Veit, Robertson, Hamel, & Friesen, 2005). Fort Hood is comprised of mostly contiguous habitat across 93 km^2^ while other central Texas sites are components of smaller habitat patches (ranging from 0.4 to 1.2 km^2^) that are isolated by surrounding grazing lands and human development. Habitat fragmentation has been responsible for restricting gene flow and subsequent population structuring in avian species across habitat types at comparable spatial scales (Callens et al., 2011; Kekkonen, Hanski, Jensen, Väisänen, & Brommer, 2011; Woltmann, Kreiser, & Sherry, 2012).

A signature of asymmetrical gene flow suggest source‐sink dynamics are occurring in our study system. Source‐sink dynamics are characterized by a net flow of individuals from a source population to sink population(s), source populations with population growth, and sink populations with population decline (Peery et al., 2016). Our findings corroborate the collaborative demographic study exhibiting source‐sink dynamics in this system over a 5‐year period (Walker et al., 2016). Walker et al. (2016) found populations on the SS, ER_c_, and MD study sites had an overall increasing growth rate while the remaining populations had an overall decreasing growth rate. While the demographic study answered questions about population trends in our study system, our genetic approach provided evidence for asymmetrical gene flow that would have been otherwise logistically impossible to determine for a small‐bodied migratory passerine.

Conservation efforts for the black‐capped vireo should reflect knowledge of source‐sink dynamics in Texas surrounding Fort Hood, the strong‐hold for the species. Small, isolated populations, such as those of the central Texas sites, tend to be less reproductively successful due to nest parasitism and would go extinct without contributions through immigration (Diffendorfer, 1998). Sink sites are dependent on source sites for survival; therefore, Fort Hood is vital for the survival and persistence of the local metapopulation and conservation efforts should focus on protecting this population. However, we should be cautious in disregarding the potential conservation value of the putative sink populations off the military base. The central Texas populations sustain a substantial portion of black‐capped vireo individuals in the region and are important in overall metapopulation and species abundance. Land managers should consider the implications of both source‐sink dynamics and cowbird parasitism when developing conservation plans as uncontrolled parasitism rates for black‐capped vireos are unsustainable (Smith, Campomizzi, Morrison, & Wilkins, 2013). Our findings suggest that habitat can cowbird management on Fort Hood, might benefit surrounding sink populations by creating migrants that disperse to the central Texas sites. It is possible that some black‐capped vireo sink populations could be altered to a source or stable population via brown‐headed cowbird control (Walker et al., 2016). Future research and conservation efforts should investigate population dynamics in Oklahoma. Oklahoma populations of black‐capped vireos may also exhibit source‐sink dynamics surrounding Fort Sill, as similar management and fragmentation exist in this part of the species’ range. Given that habitat fragmentation is unlikely to halt or decelerate in the future, efforts to understand and maintain connectivity among black‐capped vireo populations will become increasingly pertinent.

In conclusion, the present study inferred dispersal from Fort Hood to central Texas populations using molecular markers. We found evidence for significant population structuring with high gene flow, consistent with gene flow patterns of a metapopulation. Moreover, through multiple methods, we consistently demonstrated asymmetrical movement from Fort Hood to central Texas sites. Our results are in concordance with the collaborative demographic data (Walker et al., 2016), further asserting that there are black‐capped vireo source‐sink dynamics in the greater Fort Hood region of central Texas. Our study system provides an empirical example of source‐sink dynamics with two lines of evidence: population growth rates (Walker et al., 2016) and movement patterns. Fort Hood populations, specifically WRc and ERc, have increasing populations producing more migrants, and thereby asymmetrical movement, to the remaining central Texas populations experiencing population declines (Walker et al., 2016). Together, our findings provide the necessary data to posit that black‐capped vireo populations in central Texas function as a source‐sink system, and point to the power of combining ecological and genetic analyses to understand the underlying structures of metapopulations.

## AUTHOR CONTRIBUTIONS

Samantha Hauser genotyped samples, analyzed data, and wrote the paper. Lauren Walker sampled black‐capped vireo blood samples, toenail clips, and pin feathers, extracted DNA from samples. Paul Leberg oversaw research and contributed to revisions to the paper.

## DATA ACCESSIBILITY

Microsatellite primers: Genbank accession numbers: EF363782 ‐ EF363795. Sampling locations and microsatellite genotypes will be available on Dryad.
